# Patient, carer and healthcare professional perspectives on increasing calorie intake in Amyotrophic Lateral Sclerosis

**DOI:** 10.1177/17423953211069090

**Published:** 2021-12-22

**Authors:** Elizabeth Coates, Nicolò Zarotti, Isobel Williams, Sean White, Vanessa Halliday, Daniel Beever, Gemma Hackney, Theocharis Stavroulakis, David White, Paul Norman, Christopher McDermott, on behalf of the HighCALS group

**Affiliations:** 1 Sheffield Clinical Trials Research Unit, School of Health and Related Research, 152640 University of Sheffield; 2 Sheffield Institute for Translational Neuroscience (SITraN), 231485 University of Sheffield, Sheffield, UK; 3 Department of Psychology, 7315 University of Sheffield, Sheffield, UK; 4 7318 Sheffield Teaching Hospitals NHS Foundation Trust, Sheffield, UK; 5 School of Health and Related Research, 15574 University of Sheffield, Sheffield, UK

**Keywords:** amyotrophic lateral sclerosis, motor neuron disease, dietetics, qualitative research, behaviour change

## Abstract

**Objectives:**

Research suggests that higher Body Mass Index is associated with improved survival in people with Amyotrophic Lateral Sclerosis (pwALS). Yet, understanding of the barriers and enablers to increasing calorie intake is limited. This study sought to explore these issues from the perspective of pwALS, informal carers, and healthcare professionals.

**Methods:**

Interviews with 18 pwALS and 16 informal carers, and focus groups with 51 healthcare professionals. Data were analysed using template analysis and mapped to the COM-B model and Theoretical Domains Framework (TDF).

**Results:**

All three COM-B components (Capability, Opportunity and Motivation) are important to achieving high calorie diets in pwALS. Eleven TDF domains were identified: Physical skills (ALS symptoms); Knowledge (about high calorie diets and healthy eating); Memory, attention, and decision processes (reflecting cognitive difficulties); Environmental context/resources (availability of informal and formal carers); Social influences (social aspects of eating); Beliefs about consequences (healthy eating vs. high calorie diets); Identity (interest in health lifestyles); Goals (sense of control); Reinforcement (eating habits); and Optimism and Emotion (low mood, poor appetite).

**Discussion:**

To promote high calorie diets for pwALS, greater clarity around the rationale and content of recommended diets is needed. Interventions should be tailored to patient symptoms, preferences, motivations, and opportunities.

## Introduction

Amyotrophic lateral sclerosis (ALS), also commonly known as motor neuron disease (MND)^1^, is a devastating neurodegenerative disorder characterised by the loss of motor neurons, which causes progressive paralysis and eventually death.^1^ Onset is typically focal (limb, bulbar, or respiratory), later spreading elsewhere in the body. Since there is no cure for ALS, treatment focuses on slowing progression and managing symptoms, including malnutrition and weight loss.^2^ When the muscles involved in swallowing are affected, it can become difficult for people with ALS (pwALS) to eat and drink enough to sustain adequate nutritional intake, thereby placing them at high risk of developing malnutrition.^2^ These issues are also exacerbated by hyper-metabolism,^3^ with resting energy expenditure being on average 20% higher than in healthy individuals.^4^

Weight loss at diagnosis is a negative prognostic indicator in ALS, and nutritional parameters typically worsen with disease progression.^5^ A population based study found that two thirds of pwALS presented with weight loss at diagnosis, with the risk of death increasing by 23% for every 10% increase in weight loss.^6^ Similarly, a recent systematic review has shown that having a higher Body Mass Index (BMI) at diagnosis is associated with greater long-term survival in pwALS.^7^ Another study showed that, among pwALS whose BMI had decreased by more than 2 points per year after diagnosis, only 10% were still alive after two years, while 60% of those whose weight had decreased by 2 points or less (or remained unchanged or increased) were still alive. These findings suggest that weight loss before or after diagnosis with ALS, is associated with shorter survival.

Therefore, increasing weight through modifying behaviours that maximise calorie intake is potentially a key therapeutic goal for healthcare professionals (HCPs) treating pwALS, particularly in the early stages of the disease. With regard to this, the use of high-calorie fatty diets in pwALS was recently tested in a placebo-controlled RCT,^8^ but there was no significant effect on weight loss or survival. However, post hoc analysis and further analysis of neurofilament light chains levels in study blood samples have suggested a survival and biological effect of a high-caloric fatty diet on pwALS with fast progressing disease.^9^ Another study evaluating support from a dietician or an mHealth app did not result in any significant differences in weight changes compared to standard care.^10^ These mixed results may be due to the oral nutritional interventions not being tailored to patients, as well as low adherence, high drop-out and study design. Thus, further research examining the psychosocial and physical barriers and enablers to increasing calorie intake in pwALS is needed to develop more effective interventions.

In addition, previous research on nutrition in ALS has mostly focussed on problems resulting from dysphagia^11^ or enteral feeding,^12^ as opposed to addressing issues with oral nutritional interventions (‘food first approach’) which typically comes before enteral feeding.^13^ One qualitative study has investigated psychosocial issues related to dysphagia for pwALS from the caregiver perspective,^14^ reporting how dysphagia can change mealtime experiences of caregivers and pwALS due to fear of choking, frustration with being unable to prevent weight loss, use of avoidance as a coping strategy, and a desire to maintain normality. Another qualitative study explored the psychological factors influencing nutritional management of pwALS from the HCP perspective.^15^ This emphasised the importance of ALS-specific knowledge of nutrition, psychosocial aspects of eating and drinking, early engagement and psychological adjustment, as well as promoting perceived control over decision-making. However, these previous studies have not considered the views of patients and how they may differ to those of carers and HCPs.

Consequently, the present study aimed to provide a more comprehensive understanding of the barriers and enablers to high calorie diets in ALS, from the perspectives of pwALS, caregivers and HCPs. In addition, the present study sought to draw on the COM-B (Capability, Opportunity, Motivation, Behaviour) model of behaviour change^16^ as an overarching theoretical framework to interpret the key barriers and enablers. The COM-B model proposes that behaviour change is driven by a person's Capability (psychological or physical capability to enact a behaviour), Opportunity (the physical or social environment that enables or inhibits behaviour), and Motivation (reflective or automatic mechanisms that guide behaviour). The model has been used to identify barriers and enablers to a wide range of health-related behaviours, including physical activity in overweight and obese pregnant women^17^ and various studies looking at nutritional behaviours.^18–20^ The Theoretical Domains Framework (TDF)^21^ represents an elaboration of the COM-B components into 14 domains, each reflecting a different influence on behaviour: (1) knowledge, (2) skills, (3) social/professional role and identity, (4) beliefs about capabilities, (5) optimism, (6) beliefs about consequences, (7) reinforcement, (8) intentions, (9) goals, (10) memory, attention, and decision processes, (11) environmental context and resources, (12) social influences, (13) emotion, and (14) behavioural regulation. Together, the COM-B and the TDF provide a theoretical framework to understanding factors that could be targeted in oral nutritional interventions to promote increased calorie intake in pwALS. The adoption of these frameworks was deemed important in light of their successful implementation in previous research around nutritional behaviours in several populations,^18–20^ as well as to facilitate the development of a new intervention to support increased calorie intake by people with ALS.

## Methods

### Setting and design

A multi-centre qualitative study was carried out in eight MND centres in secondary care National Health Service (NHS) hospitals in the UK. HCPs from all centres took part in focus groups, while patient and carers were recruited from five of these centres for individual or joint interviews.

### Eligibility criteria

HCPs involved in nutritional management of pwALS were eligible to take part, including medics, specialist nurses, dietitians, speech and language therapists (SLTs), physiotherapists, occupational therapists (OTs), and psychologists. Patients were eligible to participate if they were aged 18 years or over and had received a diagnosis of ALS. Additional inclusion criteria included the progressive muscular atrophy variant where appropriate investigation excluded mimics of ALS, clinician judgement indicating suitability of patient to take part, and capacity to give informed consent and fluency in English. Patient exclusion criteria were: co-morbidity that would affect survival or metabolic state (e.g. unstable thyroid disease or diabetes mellitus) and BMI ≥ 35 kg/m2. Anyone acting as one of the main carers for pwALS was eligible to participate.

### Sampling technique

The eight NHS MND centres were sampled purposively based on geographical location, hospital size, and service configuration. Within each centre, individual staff were recruited for focus groups (FGs) using convenience sampling to reflect team composition, staff availability, and willingness to participate. Where feasible, the objective was to achieve representation from all clinical disciplines present in local teams, with up to a maximum of eight participants in each FG. Patients were sampled purposively from five of these centres to capture variation in terms of age, gender, time since diagnosis, carer presence, stage of the disease, and presence of eating and drinking difficulties.

### Recruitment

Local gatekeepers in each centre made the initial approach to HCPs by introducing the study during a staff meeting or via email. Staff members could opt-in by contacting the research team directly or providing consent for the gatekeeper to pass on contact details. The research team then contacted all volunteers to provide them with more study information and FG logistics.

Clinical staff from participating centres identified and recruited patients via current caseloads, clinics, and local advertising. Patients were asked to opt-in by contacting the research team directly or giving consent for the clinical team to pass on contact details. Members of the research team then contacted potential participants to provide more information and confirm eligibility. Following confirmation of eligibility and based on the purposive sampling strategy, patients and carers were contacted to organise a mutually convenient time and place for the interview. Where participants had significant communication difficulties, they were sent a communication support plan to complete, to ensure that they were adequately supported during interviews. A simplified version of the interview schedule was also sent to all participants beforehand.^22^

### Ethics

Ethical approval for this study was granted by the North West – Greater Manchester East NHS Research Ethics Committee (ref: 18/NW/0638), and governance approval was granted by the Health Research Authority (ref: 250732). Access to research sites was granted via local NHS Research and Development departments. All participants provided informed consent to take part.

### Participants and procedure

In total, 51 HCPs took part in eight FGs that were conducted across the eight MND centres (see Table 1). The number of participants in each FG varied from five to nine, and the composition of each varied in line with local team membership and their availability to participate. Discussions were completed face-to-face on NHS property sites convenient to participants. The median length of the focus groups was 62 min (58–75 min). See details of FG discussion guide in Supplementary Material 1.

**Table 1.** Number and specialism of focus group attendees.

Focus group	Participants	Specialism attended									
		Doctor	Nurse	Dietitian	Speech and Language Therapist	Occupational Therapist	Physiotherapist	Community Outreach	Psychology	Care Co-ordinator	
1	7	1	1	1	1	1	0	1	1	1	
2	7	1	3	1	1	0	1	0	0	0	
3	9	1	1	3	2	1	0	0	0	1	
4	6	0	1	4	1	0	0	0	0	0	
5	5	3	1	1	0	0	0	0	0	0	
6	6	0	2	2	0	0	1	0	0	1	
7	5	0	0	1	1	1	0	0	0	1	
8	6	2	1	2	0	0	0	0	0	1	
**Total**	**51**	**8**	**10**	**15**	**6**	**3**	**2**	**1**	**1**	**5**	

Thirty-four pwALS and carers were interviewed, comprising 20 individual interviews (eleven patients and nine carers) and seven joint interviews. These took place face-to-face at their homes or on hospital premises. The median length was 40 min (25–60 min) for individual interviews and 71 min (59–81 min) for joint ones. Details of the interview schedule are in Supplementary Material 2. The recruitment flow chart is provided in Supplementary Material 3.

Table 2 outlines the demographic and nutritional characteristics of pwALS (*n* = 18). Sixty-one percent of participants were male, median 67 years old, and generally in their second year post-diagnosis. Two-thirds (67%) of patients reported limb onset ALS, 61% reported dysphagia and 28% reported placement of an enteral feeding (PEG: percutaneous endoscopic gastronomy) tube, although all patients were still able to take food by mouth. The majority reported reasonably good mobility (61%). Participants’ median BMI was 25 kg/m^2^, and there was a fairly even split between those with stable weight and those who experienced weight loss or gain. Most participants had received some nutritional advice, could still eat until they were satisfied, and felt that they took longer to finish their meals since diagnosis. Around half of participants reported modifying their food texture and fewer followed an enriched diet.

**Table 2.** Demographic and nutritional characteristics of patients (N = 18).

Characteristic	Value
Median age (min-max)	67.0 (40.0-75.0)
Female N (%)	7 (38.9)
Median months since diagnosis (min-max)	17.0 (11.0-84.0)
ALS onset N (%)	
*Limb*	12 (66.7%)
*Bulbar*	6 (33.3%)
Reported dysphagia N (%)	11 (61.1%)
PEG placement N (%)	5 (27.8%)
Reported comorbidities N (%)	3 (16.7)
Reported mobility N (%)	
*Good*	11 (61.1)
*Average*	3 (16.6)
*Poor*	4 (22.2)
Median Body Mass Index (min-max)	24.7 (15.70-28.0)
Weight change (%)	
*Reduced*	6 (33.3)
*Stable*	7 (38.9)
*Increased*	5 (27.8)
Nutritional intervention (%)	
*Has received support/advice*	14 (77.8)
*Follows an enriched diet*	7 (38.8)
*Modifies food texture*	8 (44.4)
*Takes longer to finish meals*	11 (61.1)

All data were collected between 11 December 2018 and 7 March 2019. Data collection stopped when data saturation was reached. All interviews and focus groups were conducted by NZ, IW and EC, were recorded using a digitally encrypted recording device, and transcribed verbatim.

**Data analysis** Data were analysed with a template analysis approach,^23^ using NVivo® software to support data processing. For the purposes of analysis, the dataset was divided into (1) FGs with HCPs and (2) patient and carer individual and dyad interviews. The template analysis was completed by EC, NZ and IW, following the six key stages, i.e., (1) familiarisation with the datasets by reviewing all the transcripts; (2) identifying preliminary codes in the data and (3) organising them into a hierarchy or structure, and then using this to (4) develop an initial coding template, which was then (5) applied to the dataset and developed iteratively to lead to the last stage (6) where the template was finalised and applied across the dataset.

Following completion of the template analysis and identification of codes for both datasets, the codes were deductively mapped to the TDF^21^ and COM-B model^16^ by PN (and double mapped by EC) to help structure the interpretation of the identified barriers and enablers.

## Results

Fifty-three codes regarding barriers and enablers to increasing calorie intake in pwALS emerged from both datasets (Table 3). These are presented below, structured around the COM-B model components and TDF domains. Specific codes are highlighted in *italics* and illustrated with quotes from HCPs, pwALS (P) and carers (C) given in Supplementary Material 4.

**Table 3.** Barriers and enablers to increasing calorie intake for people with ALS, mapped to the COM-B model and TDF.

COM-B Component	TDF Domain	Code	Barrier or enabler	P/Cs	HCPs
Capability - Physical	Physical (skill)	Swallow/dysphagia	B	✓	✓
		Chewing	B	✓	✓
		Weakness and fatigue	B	✓	✓
		Capacity to eat	B	✓	✓
		Capacity to cook or shop	B	✓	✓
		Choking or aspiration	B	✓	✓
	N/A (Disease characteristic)	Weight	B	✓	✓
		Body shape changes	B	✓	✓
		Taste	B	✓	✓
		Salivation or secretions	B	✓	✓
		Heterogeneity of changes	B	✓	✓
		Mobility	B	✓	✓
		Breathing	B	✓	✓
		Lip seal	B	✓	
		Other health needs	B		✓
Capability - Psychological	Knowledge	Knowledge about ‘healthy eating’	B&E	✓	✓
		Knowledge about high calorie diets	B&E	✓	✓
		Lack of guidance	B		✓
	Memory, attention and decision processes	Cognitive difficulties	B	✓	✓
		Comprehension of healthcare professional advice or support	B&E	✓	
		Overwhelmed at diagnosis	B		✓
Opportunity – Physical	Environmental context/resources	Availability of informal carers or support	B&E	✓	✓
		Availability of formal carers or support	B&E	✓	✓
		Availability of peer support network	B&E	✓	
		Availability of food	B&E	✓	
		Availability of healthcare professional advice or support	B&E	✓	✓
		Lack of care continuity and geographical variation	B		✓
Opportunity - Social	Social influences	Social aspects of eating	B	✓	✓
		Influence of informal carers or support	B&E	✓	✓
		Influence of formal carers or support	B&E	✓	✓
		Influence of peer support network	B&E	✓	
		Influence of healthcare professional advice or support	B&E	✓	✓
		Delivery of person centred care	E		✓
		Building relationships with patients	E		✓
Motivation – Reflective	Beliefs about consequences	Beliefs about healthy eating	B&E	✓	✓
		Beliefs about high calorie diets	B&E	✓	✓
	Identity	Interest in healthy lifestyles	B	✓	✓
		Acceptance of and adjustment to diagnosis	B&E	✓	✓
	Goals	Body weight goals	B	✓	
		Adherence / receptivity to healthcare professional advice	B&E	✓	✓
		Sense of control	B&E	✓	✓
		Independence	B&E	✓	✓
		Patient priorities (i.e. not food)	B		✓
		Importance of patient choice and compromise	B&E		✓
	Optimism	Living in the present	E	✓	✓
		Uncertainty about future	B	✓	✓
Motivation – Automatic	Reinforcement	Eating habits and routines	B&E	✓	✓
	Emotion	Appetite and thirst	B	✓	✓
		Food enjoyment	B&E	✓	✓
		Resistance and denial	B	✓	✓
		Low mood	B	✓	✓
		Embarrassment	B	✓	✓
		Carer burden	B	✓	✓

### Physical Capability

#### Physical skills and disease characteristics

A number of physical changes were identified by pwALS, carers, and HCPs as barriers to increasing calorie intake. These included *swallowing difficulties (dysphagia)* causing difficulties with particular food textures; *breathing difficulties* which may increase the risk of *aspiration or choking*; general *muscle weakness, fatigue* and reduced *mobility*, which can affect the *conduct of everyday nutritional behaviours*, such as shopping, cooking, handling cutlery, eating, or sitting upright. Other physical changes included excessive *salivation and secretions*, *chewing difficulties*, issues with *lip seal*, loss of *sense of taste*, *weight loss* and *body shape changes*.

HCPs consistently highlighted the *heterogeneous nature of the physical changes caused by ALS*. This reflects the characteristics of different disease onsets, but also the heterogeneity of symptoms and disease course, which can exacerbate the challenge of initiating nutritional interventions in a timely fashion.

### Psychological Capability

#### Knowledge

Patients’ and carers’ knowledge about food and nutritional behaviours served as both a barrier and enabler to increasing calorie intake of pwALS, with the contrast between individual *knowledge of healthy eating* and *ALS recommended diets* being difficult for many to reconcile.

This was more of an issue for those participants with a greater interest in healthy lifestyles (see ‘Reflective Motivation’). For others, there was a willingness to increase the calorie value of their diets in line with HCP advice, albeit amidst confusion about the appropriateness of this approach. HCPs consistently demonstrated their knowledge of the potential benefit of high-calorie diets for pwALS, and explained the rationale for this approach, relating it to their knowledge of current research.

In contrast, some HCPs critiqued the evidence and, despite their support for increasing calories, they were keen to convey both its strengths and weaknesses during patient consultations. Related to this, HCPs reported a general *lack of guidance* specific to the nutritional management of pwALS. Although NICE guidelines (both MND care and nutrition support) and MND Association standards for care were mentioned, many HCPs still relied on professional experience to inform practice. Whilst HCPs were typically confident about their level of expertise, not directly identifying this as a barrier, the differences in care caused by a lack of clear guidance were acknowledged (see ‘Physical Opportunity’), especially in terms of addressing potential unhelpful beliefs.

#### Memory, attention and decision processes

Another challenge identified by HCPs related to *cognitive difficulties* affecting some patients’ *capacity to comprehend* dietetic advice alongside all of the other healthcare advice imparted to them, particularly if they are *overwhelmed at diagnosis*. HCPs were conscious of how overwhelming the time post-diagnosis can be, and the challenges of educating patients about high-calorie diets when their priorities may lie elsewhere.

### Physical Opportunity

#### Environmental context/resources

The *presence of informal carers* (partners, family members, friends) was another important enabler of nutritional behaviours identified by HCPs, pwALS, and carers themselves. Where available, support from informal carers was important to the *availability of food* and conduct of shopping, cooking and eating, although this sometimes came at the cost of carer burden (see ‘Automatic Motivation’). As a potential solution for some patients, *formal carers* (including visiting care assistants, nurses, or live-in carers) provided other essential sources of support.

However, for pwALS who did not have access to family or peer support, and for whom formal care or living in a care home are the only options, the caring experience may vary considerably based on income or financial availability, especially in terms of control over nutritional choices.

More generally, some pwALS and their carers also highlighted the importance of a wider *peer support network* and how this can help facilitate oral nutritional behaviours.

Notwithstanding the difficulties posed by the contrast between healthy eating and ALS dietary advice, the *availability of healthcare professional advice and support* was key to promoting or impeding behaviours to increase calorie intake. Some patients reported that they had not received any advice or were unable to recall this, and that they felt unsupported. This issue was corroborated by HCPs, who recognised the challenges of delivering timely, person-centred nutritional care within a ‘window of opportunity’ (FG3, FG4), given the speed of deterioration of ALS: an issue which was compounded in those centres with inadequate funding for staff time. This issue was exacerbated by the time-consuming nature of ALS care, for which a typical appointment duration was described as insufficient.

HCPs also identified several other issues that *impede continuity of care* and create *geographical variation* in services offered. These included inconsistent team composition (absence of dietitian), variation in funding available for nutritional management interventions (adaptive cutlery, oral nutritional supplements), limited specialist knowledge beyond the multi-disciplinary team (e.g. non-specialist neurological departments, GPs or community services), or the presence of special interest in ALS by staff, which could act as both a strength and limitation of teams. The passion and commitment to ALS of some HCPs was duly recognised, but so was the vulnerability of the staffing arrangement.

### Social Opportunity

#### Social influences

Corresponding with their availability, the *influence of informal or formal carers* was another major influence (both positive and negative) on nutritional behaviours identified by all participants. Specifically, the wider *peer support network* was identified as a facilitator of social opportunities for eating and drinking by pwALS and carers.

More generally, the *social aspects of eating* and drinking, such as socialising, eating together as families or visiting restaurants and cafes were highlighted during all FGs. HCPs spoke about the loss they see for pwALS and their families as the disease progresses. Carers and pwALS also explained how disease progression can hamper the *social aspects* of eating, such as meal sharing or eating out.

The influence of *health professional advice and support* was also key for pwALS, despite varied levels of receptivity and acceptance of the diagnosis (see ‘Reflective Motivation’). Adopting a proactive approach to nutrition was often described by HCPs as another enabling aspect of *patient-centred care* for pwALS. This included the chance to offer dietetic and speech and language therapy advice before developing problems with eating and drinking, as well as undertaking frequent home visits and sharing insights across the Multi-Disciplinary Team. Introducing ideas proactively and sensitively was believed to provide a platform for future care discussions when patients might be more ready to make changes to nutritional behaviours. Correspondingly, most HCPs spoke about the facilitative value of *building relationships with patients* and their families to help understand priorities as well as promoting patient choice and compromise.

### Reflective Motivation

#### Beliefs about consequences

A major aspect which could enable or inhibit changes to nutritional behaviours was differing *beliefs about healthy eating versus high calorie diets* to promote good health. These were largely in line with the knowledge of pwALS, their carers, and HCPs (see ‘Psychological Capability’). However, when in conflict, reconciling them could prove challenging, making it difficult for pwALS and their carers to adopt higher calorie diets.

#### Identity and goals

Increasing calorie intake was often seen as problematic for those pwALS who used to be very active and for whom living a ‘*healthy lifestyle’* and meeting desired *body weight goals* was previously an important part of their identity.

HCPs also reported a range of patient responses to being encouraged to consume more calories, from the positive, to the confused or resistant, and they related this to patients’ perspectives on *healthy lifestyles*.

Despite some positive feedback of promoting high calorie diets, HCPs in each FG also spoke about the challenges of encouraging *adherence* to high-calorie diets by pwALS who have strong beliefs about *healthy lifestyles*. They also spoke about the facilitative potential of *patient choice and compromise* by working closely with them to understand their *priorities*, knowledge, and beliefs about food.

Many patients and carers were keen to express their *receptivity to advice* and support from HCPs, but this varied considerably among participants. The contrast between ‘healthy eating’ and high calorie dietary advice for pwALS, or the absence of regular or timely support, were influential factors.

Another theme related to pwALS’ *acceptance of the ALS diagnosis* and lack of psychological adjustment to the need to change nutritional behaviours, which presented a potential barrier to achieving a high calorie diet. Conversely, others talked about feelings of *control* and *independence*, and how exercising agency or decisional input over some aspects of nutrition may be an empowering experience, at least whilst they were still able to maintain their independence.

#### Optimism

Levels of *optimism* about the diagnosis and the future also influenced how pwALS approached their nutritional management. For example, where pwALS approached their illness in a more positive way or were more accepting of their condition, they were typically more keen to make changes to their diets, and vice versa.

### Automatic Motivation

#### Reinforcement

*Eating habits and routines* relating to food choices and household roles in shopping and cooking were other influences on the nutritional behaviours of pwALS. Food preferences can be very difficult to change in line with dietary advice as the disease progresses, as this can affect the whole household. Many participants described changes to roles in the maintenance of food-related activities which could prove challenging to both the pwALS and carer, regardless of their domestic roles pre-diagnosis. Relinquishing responsibility for shopping and cooking could be upsetting to pwALS regardless of gender, as well as a source of tension in relationships.

#### Emotion

The physical changes that pwALS experience can also impact on *appetite and thirst*, and impede efforts to maintain a high calorie intake. Physical symptoms also impacted on the *enjoyment of food and drinks*. Although many pwALS were able to continue eating and drinking as usual, others found this less enjoyable over time, and this was a source of loss.

Some participants also talked about feelings of *resistance and denial,* from both patients and carers, which could interfere with increasing calorie intake. HCPs also noted how challenging it can be to engage more resistant individuals.

*Low mood*, coupled with the *embarrassment* of challenges with eating or drinking in public or social settings was identified as another barrier to achieving high calorie diets.

Finally, emotional responses from spouses, family members, and friends – and the *carer burden* of providing long-term support to pwALS – were also mentioned as barriers to changing eating behaviours. More specifically, asking pwALS to change their dietary intake and seeing the person deteriorate can place a substantial burden on informal carers, triggering low mood or anxiety, which in turn could limit their capacity to support changes to food and drink.

## Discussion

### Summary of main findings

This study identified a complex range of factors that influence the uptake of high calorie diets among pwALS. To our knowledge, this is the first qualitative investigation to specifically triangulate the views of pwALS, carers, and HCPs on high calorie diets in ALS. The use of the COM-B model^16^ and TDF^21^ was helpful in structuring the analysis and interpretation of barriers and enablers. To achieve high-calorie diets for pwALS, the analysis highlighted the need to address all three COM-B components and 11 TDF domains.

In relation to the ‘Capability - Physical’ component, many physical symptoms associated with the progression of ALS affect people's eating and drinking capabilities over time. In line with evidence documenting the risks of weight loss in ALS,^7^ most current research has focussed upon clinical management of these symptoms, including dysphagia^11^ or enteral feeding.^12^ Whilst it is important not to diminish the impact of the physical progression of the disease, our analysis also highlighted the importance of social and psychological influences on eating and drinking behaviours in ALS.

The ‘Capability – Psychological’ component captured the importance of knowledge about healthy eating and high calorie diets. Whilst some pwALS were keen to increase their intake as they enjoyed calorie-dense foods, others struggled to make those changes due to concerns about the impact on general physical health, influenced by pre-existing views about healthy eating. Despite some evidence that high calorie diets may be beneficial, along with considerable support for this approach amongst HCPs in this study, the limits of the evidence base and a lack of specific supporting guidance represents a challenge for current practice. This finding is consistent with recent evidence charting the provision of ALS nutritional management with HCPs.^15, 24^

Within the ‘Opportunity – Physical’ and ‘Opportunity – Social’ components, the availability and influence of both formal healthcare provision and informal caring networks were highlighted. A lack of care continuity and geographical variation, and the facilitative power of person-centred care, have also previously been documented in a survey of ALS HCPs.^24^ The importance of case management in ALS has also recently been highlighted.^25, 26^ Similarly, the significant role played by informal carers in ALS is well documented,^27, 28^ but our study sheds light on how they contribute in relation to eating and drinking.

Considering the ‘Motivation – Reflective’ and ‘Motivation – Automatic’ components, this study also identified a number of psychological factors. These included beliefs about healthy eating and high-calorie diets, the varied importance of pre-diagnosis identity and lifestyle, as well as adjustment to diagnosis and engagement with healthcare interventions. Moreover, pwALS, their carers, and HCPs drew attention to the impact of different goals, levels of optimism, existing habits, and routines on behaviour. Finally, a number of emotional barriers to changing behaviour, such as emotional burden upon carers and low mood, resistance, denial, and embarrassment amongst pwALS were documented. Whilst new within the context of psychosocial issues related to eating and drinking, these findings are consistent with previous evidence on the general impact of dysphagia in pwALS.^14, 15^ Likewise, there is a body of work which considers the psychological burden of ALS on both people with the disease and carers.^28^

### Strengths and limitations

This is the first study to provide an in-depth exploration of the barriers and enablers to increasing calorie intake in pwALS within a relatively large qualitative sample of patients, carers, and HCPs in the UK. This will aid the development of effective interventions to prevent weight loss and potentially increase survival in pwALS.

Nonetheless, this study has some limitations. In particular, the self-selecting sample may have introduced some biases. It is possible that we have not accurately captured the impact of some aspects, such as patient shock at diagnosis and disengagement from healthcare provision, as those pwALS are unlikely to participate in research. Nonetheless, participants highlighted a wide range of factors affecting dietary behaviour. Moreover, the insights of HCPs, who spoke at length about the challenges of changing eating behaviour in resistant or disengaged patients, may also give confidence that a full range of important barriers were identified (notwithstanding the possibility that disengaged patients may have different views about these issues to HCPs). Finally, while it is possible that we mainly captured the perspectives of the HCPs most engaged with ALS nutritional care, as the HCPs were self-selecting, we nonetheless recruited a broadly representative sample of staff involved in the nutritional management of pwALS.

### Implications for clinical practice

Our results clearly outline the need for nutritional management interventions for pwALS which can be tailored in order to accommodate differing symptoms, knowledge, beliefs, and nutritional preferences, as well as the environmental contexts/resources and social influences at play. In tailoring interventions, HCPs must also consider where pwALS are psychologically – in terms of acceptance of the diagnosis, adjustment to symptoms, and other emotional responses^29^ – because, without this wider perspective, our findings suggest that the effectiveness of interventions will be restricted. Future research should therefore focus on developing and evaluating complex interventions that are able to address the wide range of barriers in the oral nutritional management of pwALS. We believe our findings provide a valuable starting point in this direction.

## Supplemental Material

sj-docx-1-chi-10.1177_17423953211069090 - Supplemental material for Patient, carer and healthcare professional perspectives on increasing calorie intake in Amyotrophic Lateral Sclerosis Click here for additional data file. Supplemental material, sj-docx-1-chi-10.1177_17423953211069090 for Patient, carer and healthcare professional perspectives on increasing calorie intake in Amyotrophic Lateral Sclerosis by Elizabeth Coates, Nicolò Zarotti, Isobel Williams, Sean White, Vanessa Halliday, Daniel Beever, Gemma Hackney, Theocharis Stavroulakis, David White, Paul Norman, Christopher McDermott and in Chronic Illness

sj-docx-2-chi-10.1177_17423953211069090 - Supplemental material for Patient, carer and healthcare professional perspectives on increasing calorie intake in Amyotrophic Lateral Sclerosis Click here for additional data file. Supplemental material, sj-docx-2-chi-10.1177_17423953211069090 for Patient, carer and healthcare professional perspectives on increasing calorie intake in Amyotrophic Lateral Sclerosis by Elizabeth Coates, Nicolò Zarotti, Isobel Williams, Sean White, Vanessa Halliday, Daniel Beever, Gemma Hackney, Theocharis Stavroulakis, David White, Paul Norman, Christopher McDermott and in Chronic Illness

sj-docx-3-chi-10.1177_17423953211069090 - Supplemental material for Patient, carer and healthcare professional perspectives on increasing calorie intake in Amyotrophic Lateral Sclerosis Click here for additional data file. Supplemental material, sj-docx-3-chi-10.1177_17423953211069090 for Patient, carer and healthcare professional perspectives on increasing calorie intake in Amyotrophic Lateral Sclerosis by Elizabeth Coates, Nicolò Zarotti, Isobel Williams, Sean White, Vanessa Halliday, Daniel Beever, Gemma Hackney, Theocharis Stavroulakis, David White, Paul Norman, Christopher McDermott and in Chronic Illness

sj-docx-4-chi-10.1177_17423953211069090 - Supplemental material for Patient, carer and healthcare professional perspectives on increasing calorie intake in Amyotrophic Lateral Sclerosis Click here for additional data file. Supplemental material, sj-docx-4-chi-10.1177_17423953211069090 for Patient, carer and healthcare professional perspectives on increasing calorie intake in Amyotrophic Lateral Sclerosis by Elizabeth Coates, Nicolò Zarotti, Isobel Williams, Sean White, Vanessa Halliday, Daniel Beever, Gemma Hackney, Theocharis Stavroulakis, David White, Paul Norman, Christopher McDermott and in Chronic Illness

## Notes

1. ALS is the internationally recognised term for this disease, but Motor Neuron Disease (MND) is the preferred term in the UK. ALS is one type of MND. ALS is applied in this paper except where MND is used by participants or to refer to services.
