# Supplementary material for: Patient, carer and healthcare professional perspectives on increasing calorie intake in Amyotrophic Lateral Sclerosis
Source: Chronic Illn. 2021 Dec 22;19(2):368–82. doi: 10.1177/17423953211069090 (PMC9999280; doi:10.1177/17423953211069090)
Supplement: sj-docx-1-chi-10.1177_17423953211069090 - Supplemental material for Patient, carer and healthcare professional perspectives on increasing calorie intake in Amyotrophic Lateral Sclerosis [file sj-docx-1-chi-10.1177_17423953211069090.docx]

**Patient, carer and healthcare professional perspectives on increasing calorie intake in Amyotrophic Lateral Sclerosis**

**Supplementary material 1 – Focus group discussion guide**

**Context**

**1. Please tell me briefly about yourself and the work you do with people with MND /ALS? [keep this brief - just for introductions]**

Prompts

- 1. Job role
  2. Time in post

**2. Do you *routinely* provide nutritional management services to people with MND/ALS?**

**3. Please tell me about what nutritional management services are given to people with MND/ALS in this area. [Note to interviewers: keep this brief (approx. ten minutes)]**

Prompts

1. Who delivers this and where?
2. How and when are patients with nutritional needs identified?
   1. What happens after diagnosis?
   2. What routine assessments are undertaken?
   3. Do patients have to raise issues themselves (who are they referred to)?
3. Which interventions are provided in your area?
   1. Examples: food first/dietary enrichment, types of ONS, written / online support, face-to-face support from dietician etc.
4. What guidance is used to inform how nutritional management?

**4. What do you think about increasing calorie intake for people with MND / ALS?**

Prompts

1. Can you say a little more about that?
2. Do you have a clear idea about nutrition and MND/ALS?
3. If so, why do you think that is?
4. If not, what is confusing?

***Barriers and enablers***

**Service delivery**

**5. What are the main challenges to delivering nutrition and dietetic services to people with MND/ALS in your area?**

Prompts

1. Is nutritional management seen as important in your area?
2. Do healthcare professionals have the knowledge and skills to provide effective nutritional management?
3. Do you have the right staff in the team?
4. Are the services commissioned appropriately in your area?

**6. How could you overcome the barriers to delivering nutrition and dietetic services to people with MND/ALS in your area?**

Prompts

1. Could the importance of nutritional management be emphasised among healthcare professionals? How might this be achieved?
2. What would effective training for healthcare professionals look like?
3. How would the MDT be best structured to support nutritional management?
4. Are there any better ways to commission services in your area?

**7. Is there anything that do you do already that you think works well or is successful?**

***Nutritional interventions***

**8. What are the key challenges that you face when attempting nutritional management?**

Prompts

1. Do you have enough guidance on what can help patients?
2. Availability of different interventions/management strategies?
3. Acceptability of different interventions/management strategies?
4. Is it difficult to get the timing right?
5. Do patients struggle to follow your advice?
6. How easy is it for patients to implement your suggestions?
7. How do patients feel about their nutritional management?

**9. What might help you to overcome the barriers that you or your patients face when you are supporting their nutritional intake?**

Prompts

1. Would any additional literature / leaflets be helpful? What might they look like?
2. How about education / training for yourself or the patient?
3. Might different equipment / tools support you / the patient to overcome these barriers?

**10. Is there anything that do you do already that you think works well or is successful?**

***Other ways of working***

**11. If we were to advise all people living with MND/ALS to increase their calorie intake, how do you think we could best support them to do this?**

**12. If we asked patients to increase their calorie intake at diagnosis, how do you think this would work for your local area?**

Prompt

1. How do you think patients and carers would respond to being asked to increase their calorie intake at the point of diagnosis?

***Closing remarks***

**13. Is there anything else that you would like to say that you haven't had the opportunity to say yet?**
