# Supplementary material for: Patient, carer and healthcare professional perspectives on increasing calorie intake in Amyotrophic Lateral Sclerosis
Source: Chronic Illn. 2021 Dec 22;19(2):368–82. doi: 10.1177/17423953211069090 (PMC9999280; doi:10.1177/17423953211069090)
Supplement: sj-docx-2-chi-10.1177_17423953211069090 - Supplemental material for Patient, carer and healthcare professional perspectives on increasing calorie intake in Amyotrophic Lateral Sclerosis [file sj-docx-2-chi-10.1177_17423953211069090.docx]

**Patient, carer and healthcare professional perspectives on increasing calorie intake in Amyotrophic Lateral Sclerosis**

**Supplementary material 2 – Patient and carer interview schedules**

**Patients**

**Context**

1. **Please tell me briefly about yourself and your MND/ALS?**

*[Note to interviewers: this is a warm up question – need to be mindful of time and energy given to answer this- - try to keep it factual]*

Prompts

- 1. Diagnosis – process, details, time since
  2. Stage of the disease
  3. Treatments received
  4. Other healthcare received – e.g. hospital and community care
  5. Other support received

1. **What does food and eating mean to you?**

Prompts

1. What kind of food do you like?
2. Who prepares the food you eat? Who does the cooking in the house?
3. Who does the food shopping in the house?
4. How important is food and nutrition to your life?
   - Explore social aspects, current perceptions about food and weight
5. How much control do you feel with food and nutrition choices?

**Experiences**

**3.** **Do you have any issues with eating / swallowing / chewing?**

*[Note to interviewers: Prompts a and b are key questions to identify/explore key barriers and enablers - spend time of these]*

Prompts

- 1. What are the difficulties / issues?
  2. What helps?
  3. Do you get any help with eating? What does this involve?
  4. Did you experience any issues before diagnosis?

**4. Has your experience of food and eating changed since your symptoms first began?**

Prompts

1. If so, what has become more difficult (taste, appetite, chewing/swallowing, functional status, attitudes to eating, motivation to eat, ability to feed self)? What, if anything, helps?
2. If it has not become more difficult, can you say a little more about that?

**5. Have you received any support or advice for food and eating since your symptoms first began?**

Prompts

*[Note to interviewers: need to tailor this to issues experienced by participants/response to Q3/4]*

1. What kind of advice have you received?
2. When were you advised about this and by whom?
3. What did you think of this? (Like/dislike and reasons)
4. What kind of support / interventions have you been given?
   1. When were these introduced to you and by whom?
   2. What do you think of this? (Like/dislike and reasons)
5. What is your experience of how your nutrition has been managed since your symptoms first began?
   1. How was the need for these interventions explained to you?
   2. Did you agree with the reasons? Did you think it was important to follow the advice given?
   3. Did you find any differences between approaches of different health professionals?
6. What helps you to keep to the advice or support given?
   1. How were you supported to keep to the advice (examples: monitoring frequency; face-to-face vs phone; use of self-monitoring; written information given; use of patient specific plans.)?
7. What challenges have you experienced following the advice given?

**Views**

*Introduction: Research suggests that people with MND/ALS need more calories and many are being advised by health professionals to eat a high calorie diet.*

**6. What do you think about increasing calorie intake in MND?**

Prompts

1. What impact, if any, do you believe increasing your calorie intake might have on your health / MND?
2. Can you say a little more about that?
3. Do you have a clear idea about the impact of nutrition on and MND/ALS?
   1. If so, why do you think that is?
   2. If not, what is confusing?
4. [For people who haven’t been asked to increase calorie intake before]: How would you feel about being asked to increase your calorie intake?

**7. How do you think you could increase your calorie intake?**

Prompts

1. What do you think are the challenges with these approaches?
2. What might help you to overcome them?

**8. How do you think we could best support you to increase your calorie intake them to do this?**

Prompts

1. What about other people with MND/ALS?

**Closing remarks**

**9. Is there anything else that you would like to say that you haven't had the opportunity to say yet?**

**Carers**

**Context**

1. **Please tell me briefly about yourself and the person in your life who has MND/ALS?**

*[Note to interviewers: this is a warm up question – need to be mindful of time and energy given to answer this - try to keep it factual]*

Prompts

- 1. What is your relationship to this person?
  2. Diagnosis – process, details, time since
  3. Stage of the disease
  4. Living arrangements
  5. How do you care for this person?
  6. Other support received (formal carers, healthcare professionals)

**2. What does food and eating mean to you?**

Prompts

1. Who does the cooking in the house?
2. Who does the food shopping in the house?
3. How important is food and nutrition to your life?
   1. Explore social aspects, current perceptions about food and weight
4. How much control do you feel with food and nutrition choices?

**Experiences**

**3. Does [person with MND/ ALS] have any issues with eating / swallowing / chewing?**

Prompts

1. What are the difficulties?
2. What helps?
3. Do you give them any help with eating? What does this involve?

**4. Has your experience of food and eating with [person with MND/ALS] changed since their symptoms first began?**

Prompts

1. If so, what has become more difficult (taste, appetite, chewing/swallowing, functional status, attitude to eating, motivation to eat, ability to feed self)? What, if anything, helps?
2. If things have not become more difficult. If not, can you say a little more about that?

**5. Has [person with MND /ALS] received any support or advice for food and eating since their symptoms first began?**

*[Note to interviewers: need to tailor this to issues experienced by participants/response to Q3/4]*

Prompts

1. What kind of advice has been received?
    i. When was this advice given and by whom?
    ii. What did you think of this (like/dislike and reasons)?
    iii. How did the person with MND/ALS respond to this?
2. What kind of support / interventions have been received?
    i. When were these introduced and by whom?
    ii. What do you think of this (like/dislike and reasons)?
    iii. How did the person with MND/ALS respond to this?
3. What did you think of the advice and support the person in your life with MND/ALS has been given for eating?
    i. How was the need for these interventions explained?
    ii. Did you agree with the reasons? Did you think it was important to follow the advice given?
4. As a carer, what helps you to keep to the advice or support given?
    i. How were you/they supported to keep to the advice (e.g., monitoring frequency; face-to-face vs phone; use of self-monitoring; written information given; use of patient specific plans)?
5. As a carer, what challenges have you experienced following the advice given?

**Views**

*Introduction: Research suggests that people with MND/ALS need more calories and many are being advised by health professionals to eat a high calorie diet.*

**6. What do you think about increasing calorie intake in MND/ALS?**

Prompts

- 1. What impact, if any, do you believe increasing their calorie intake might have on their health / MND?
  2. Can you say a little more about that?
  3. Do you have a clear idea about the impact of nutrition on and MND/ALS?
     1. If so, why do you think that is?
     2. If not, what is confusing?
  4. [For people who haven’t been asked to increase calorie intake before]: How do you think the person in your life with MND/ALS would feel about being asked to increase their calorie intake?

**7. How do you think you could increase calorie intake in people with MND/ALS / how do you already try to increase calorie intake for the person in your life with MND/ALS [use the person’s name]?**

- 1. What do you think are the challenges with these approaches?
  2. What might help people with MND/ALS to overcome them?

**8. How do you think we could best support people living with MND / ALS to increase their calorie intake?**

Prompts

1. How do you think you and the person in your life with MND/ALS would have responded to these suggestions?

**Closing remarks**

**9. Is there anything else that you would like to say that you haven't had the opportunity to say yet?**
