# Supplementary material for: Patient, carer and healthcare professional perspectives on increasing calorie intake in Amyotrophic Lateral Sclerosis
Source: Chronic Illn. 2021 Dec 22;19(2):368–82. doi: 10.1177/17423953211069090 (PMC9999280; doi:10.1177/17423953211069090)
Supplement: sj-docx-3-chi-10.1177_17423953211069090 - Supplemental material for Patient, carer and healthcare professional perspectives on increasing calorie intake in Amyotrophic Lateral Sclerosis [file sj-docx-3-chi-10.1177_17423953211069090.docx]

**Patient, carer and healthcare professional perspectives on increasing calorie intake in Amyotrophic Lateral Sclerosis**

**Supplementary material 3 – Recruitment flow chart**

30 patients identified

26 patients screened

22 patients eligible

18 patient interviewed

34 participants consented and interviewed

4 patients dropped out

4 patients ineligible

16 carers interviewed

*Figure 1 -* Recruitment flowchart for patients and carers

4 patients not pursued
