# Supplementary material for: Patient, carer and healthcare professional perspectives on increasing calorie intake in Amyotrophic Lateral Sclerosis
Source: Chronic Illn. 2021 Dec 22;19(2):368–82. doi: 10.1177/17423953211069090 (PMC9999280; doi:10.1177/17423953211069090)
Supplement: sj-docx-4-chi-10.1177_17423953211069090 - Supplemental material for Patient, carer and healthcare professional perspectives on increasing calorie intake in Amyotrophic Lateral Sclerosis [file sj-docx-4-chi-10.1177_17423953211069090.docx]

**Patient, carer and healthcare professional perspectives on increasing calorie intake in Amyotrophic Lateral Sclerosis**

**Supplementary material 4 – Table of quotes to illustrate codes by COM-B component and TDF domain**

| **COM-B Component** | **TDF Domain** | **Code** | **Quote(s)** |
| --- | --- | --- | --- |
| Capability – Physical | Physical (skill) | Swallow/dysphagia | ‘That is hard, as I can’t chew and swallow, and have to use cloth to put over mouth. My chewing is odd because my tongue does not work to stir food in mouth and deliver to throat to swallow.’ (P9)  Sometimes if respiratory issues are a problem as well the sheer coordination of breathing, swallowing, chewing, can be quite difficult.’ (Community Dietitian, FG3) |
|  |  | Chewing |  |
|  |  | Weakness and fatigue |  |
|  |  | Capacity to eat |  |
|  |  | Capacity to cook or shop |  |
|  |  | Choking or aspiration |  |
|  | N/A (Disease characteristic) | Weight | ‘Crumbly things in, it’s not the swallowing, […] it’s the phlegm and the saliva that gets, everything gets caught in his throat because of that, and that makes it difficult to eat.’ (C30)  ‘I think because MND can you know manifest itself in so many different ways can’t it? I mean we had somebody who was very, very dependent on having, you know, respiratory support that actually could eat fairly well. So never had a PEG tube.’ (Community Nurse, FG6) |
|  |  | Body shape changes |  |
|  |  | Taste |  |
|  |  | Salivation or secretions |  |
|  |  | Heterogeneity of changes |  |
|  |  | Mobility |  |
|  |  | Breathing |  |
|  |  | Lip seal |  |
|  |  | Other health needs |  |
| Capability - Psychological | Knowledge | Knowledge about ‘healthy eating’ | ‘Modern day culture is to reduce calories, not increase.’ (P25)  ‘I don’t know, […] my only worry would be that we’re substituting one problem for another, in that we’re substituting weight loss with too much fat in the body, and you’re gonna die of a heart attack instead. […].’ (C20)  ‘That’s what we’re taught’ (P19) |
|  |  | Knowledge about high calorie diets | ‘The research that says generally people who have an average, or above average, BMI, basically do better than people that are on the low average BMI. And you know, we didn’t do the research, but that’s what it says. So that’s what you do.’ (MND Nurse 1, FG2)  ‘We know that it is better for them if they stay nourished for as long as they can… so I suppose we go for that, but still there isn’t any research to say that yes definitely this is the way we have to go.’ (Dietitian, FG1) |
|  |  | Lack of guidance | ‘That’s where we’re on a sticky wicket, cos you don’t really have a good evidence base, other than established practice, you know […] we seem to chuck down calories, it seems to slow down your weight loss. (Dietitian, FG4) |
|  | Memory, attention and decision processes | Cognitive difficulties | ‘Sometimes they’re so shell shocked about their diagnosis… so leave it to for the community dietitian to pick up, you know, rather than do everything within three days, it’s easier to have that in a couple of weeks.’ (MND Nurse, FG4) |
|  |  | Comprehension of healthcare professional advice or support |  |
|  |  | Overwhelmed at diagnosis |  |
| Opportunity – Physical | Environmental context/resources | Availability of informal carers or support |  |
|  |  | Availability of formal carers or support | ‘I’m fortunate to have a live-in carer. So, you know, if I can’t use, I can usually feed myself in the mornings, but basically, I’ve got someone who can feed me, so, you know, in a good place compared to many others.’ (P13)  ‘The nursing home just didn’t seem able to cater for his swallow and that’s another thing, he was just losing weight cos he couldn’t eat the food despite talking to the kitchens and, […] so he just had a PEG.’ (MND Nurse, FG5 |
|  |  | Availability of peer support network | ‘As soon as my friends knew that I was not well, they’ve all without exception said lets go and have lunch, so I’ve had lunch with lots of friends and most weeks we go out to lunch now which we never did in our lives before.’ (P45) |
|  |  | Availability of food |  |
|  |  | Availability of healthcare professional advice or support | ‘Not in the last four and a half years has anyone made any suggestions in specifics. […] It just seems to be our own common sense about what we eat. You know. We could have been eating a really crap diet, honestly, and nobody probably would have said anything, you know.’ (C2) |
|  |  | Lack of care continuity and geographical variation | ‘It’s the variability even within a team… so one of our local teams might have had somebody who was very interested in MND, so they religiously saw them every month, but then that person leaves and there’s no… it’s the lack of continuity of care and consistency of standard that is a big barrier.’ (Speech and Language Therapist, FG8) |
| Opportunity - Social | Social influences | Social aspects of eating | ‘Must be an impact on them wanting to go out to enjoy meals or go out to a normal restaurant because they might need to have added things in their meals, they would normally just eat a curry. […] All of these little things that people anticipate and it then becomes a barrier and you’ve got a whole new issue, with mental health.’ (OT, FG3)  ‘We all just sit there and we have a meal together and talk about the day, and suddenly that kind of thing’s almost been taken away from you…’ (Community Nurse, FG6)  ‘That’s a part of going out as well too, isn’t it? If you’re eating outside, you enjoy where you’d want to eat. And you lose that too.’ (P10)  ‘We don’t go out as much as we used to, eating out. We sort of have to be a little bit selective to make sure that she can, places we visit she can get in, in and out easily enough.’ (C34) |
|  |  | Influence of informal carers or support |  |
|  |  | Influence of formal carers or support |  |
|  |  | Influence of peer support network | ‘What’s kept him going the last year, the fact that we can still get him out with his mates even though it’s a struggle, and they’re all brilliant with him.’ (C18) |
|  |  | Influence of healthcare professional advice or support | ‘The ability just to very quickly build up a rapid rapport with someone and their family and to be able to build that trust almost instantly is really key and core skills, and then you’re going where you wanna take it.’ (Community Dietitian 1, FG6) |
|  |  | Delivery of person centred care |  |
|  |  | Building relationships with patients |  |
| Motivation – Reflective | Beliefs about consequences | Beliefs about healthy eating | ‘I think for optimum health you should be eating a balanced diet in terms of fruit, veg, you know, carbohydrate, protein and if you eat that and you felt in the past you’d eaten it, how you felt it was normal for you and to continue doing that would be a benefit, you know, much more enjoyable really.’ (P11)  ‘How do you up someone’s calories but keep them healthy keep them fit, especially if he can’t exercise as much as he used to we’re already noticing that he can’t exercise as much as he used to because he gets fatigued he’s just gonna get heavy he’s gonna put on weight.’ (C32) |
|  |  | Beliefs about high calorie diets |  |
|  | Identity | Interest in healthy lifestyles | ‘I spent most of my adult life either dieting, trying to eat healthy… it’s been, it’s been a big factor, trying to eat healthy, banning cakes. And now, suddenly given a bit of a green light to eat anything as long as it’s high in calories and so, I put loads of weight on like my tummy and hated every bit of it.’ (P19)  ‘It’s almost in a way counter to what they believe.’ (Community Dietitian, FG3) |
|  |  | Acceptance of and adjustment to diagnosis | ‘A lot of people diagnosed with MND suddenly shut off from wanting to use the internet and look at anything that might give them bad news, or whatever. I think there’s a lot of people like me, a lot of people that don’t, that don’t wanna know, don’t wanna even mention MND.’ (P19)  ‘It can happen at any stage. You know, quite often MND is a diagnosis that is difficult to come to terms with emotionally, psychologically.’ (Community Dietitian, FG6) |
|  | Goals | Body weight goals |  |
|  |  | Adherence / receptivity to healthcare professional advice | ‘I am very accepting of the intention, good intentions, of healthcare professionals. When they give me advice as the solution to a problem, I stick with it.’ (P15)  ‘It’s a matter of finding a balance of, there’s lots of guidance saying: we don’t want you to lose weight. There’s very little that says how we want you to stop this weight loss or slow this weight loss down. […] You’re not really given advice on that. As long as they can see the scales saying the same.’ (P19) |
|  |  | Sense of control | ‘I don’t have as much control, because I can’t go and get the foods that I want to buy. And unless I write a list with specifics, I don’t get the foods that I would probably choose myself if I went shopping, so I don’t have as much control. […] It’s just… well I’ve always been very independent and done everything for myself, and to suddenly step back and let other people do it, I find it really frustrating.’ (P43) |
|  |  | Independence |  |
|  |  | Patient priorities (i.e. not food) | ‘There’s also them ones that are particularly healthy and fit that who really struggle […] to go for the high calorie diet.’ (MND Nurse 1, FG1)  ‘You’ve got to explore their belief system around food before you start that, because otherwise you’re at a brick wall.’ (Dietitian, FG4) |
|  |  | Importance of patient choice and compromise |  |
|  | Optimism | Living in the present | ‘A lot of MND patients I’ve come across there’s not much that they have control over is there with their condition. So, some of them are trying to grip on to you know with their behaviour with like when they’re refusing or eating at risk, it’s those things that they still have control over those decisions that they can still do that might be important to them.’ (OT, FG7) |
|  |  | Uncertainty about future |  |
| Motivation – Automatic | Reinforcement | Eating habits and routines | ‘I get, quite honestly, a bit fed up with cooking, and towards this summer I really felt, I just don’t know what to cook anymore and we were having the same meals, same meals, so we’ve got to have a radical change.’ (C2)  ‘Looking at the kind of dynamics at home, cos often there was a lot of stress from a person who struggling to eat and a carer who’s trying their absolute best to get them to eat and they’re giving them mountains of food, that they then can’t eat and all that stress, that goes with that…’ (Community Dietitian, FG4) |
|  | Emotion | Appetite and thirst | ‘I do try to keep the calories up. One of the problems is that I rarely feel hungry. […] I was always hungry [before MND].’ (P6) |
|  |  | Food enjoyment | ‘It was very important! I mean it was a lot of pleasure centred around you know, food… food was a big pleasure, particularly in [patient’s] life. Wouldn’t you?’ (C30)  ‘I feel fed up sometimes seeing real food and can’t have it. Can’t get a steak in PEG. Makes me sad and I lose my flavours, it has slowly got worse over the 14 months, so now I am relaxed more having PEG, in case I can’t swallow.’ (P9) |
|  |  | Resistance and denial | ‘I’m still very much a thing of denial or fighting it, if you like. Well, at the moment, trying to say well, everything’s alright, I’m still in control. I don’t need to worry about things like that.’ (P31)  ‘I think sometimes people do think, you know, it’s not something they want to talk about, because it’s just one, when they’re trying to come to terms with the diagnosis and things like that, you know, if we don’t talk about it won’t be happening. So, there’s an element of denial there.’ (MND Nurse 1, FG2) |
|  |  | Low mood | ‘I think socially, the impact can be massive so just feeling you know, even if it’s not to do with their swallowing, just if their speech is slurred they may not want to go out and order in a restaurant […] so I think that can be massive and their mood as well, after diagnosis…’ (Speech and Language Therapist, FG7)  ‘In a restaurant, if you haven’t got the right knife or if the food is tough then, of course, that adds to it and I kind of think “I wish I hadn’t ordered this” or “I don’t really wanna eat this cause it’s just hard work”, especially in a restaurant.’ (P31). |
|  |  | Embarrassment |  |
|  |  | Carer burden | ‘I’m very, very conscious of how much he eats and making sure that he eats enough, you know, that we are getting the right calories, I think I’m a bit paranoid about weighing him.’ (C40)  ‘I think again, you know, it must be hard to, you know, I’m sure as a carer to keep setting the table for someone who’s not got a very good appetite and to keep cooking a meal and trying. And we find that a lot with carers, you know, it’s so hard…’ (Physiotherapist, FG2) |

*Note*. P = patient. C = carer. FG = focus group.
